# Gene therapies in pediatric ophthalmology

**DOI:** 10.3389/fopht.2023.1188522

**Published:** 2023-06-05

**Authors:** Alejandra Daruich, Matthieu P. Robert, Dominique Bremond-Gignac

**Affiliations:** ^1^Ophthalmology Department, Necker-Enfants Malades University Hospital, Assistance Publique-Hôpitaux de Paris (AP-HP), Paris Cité University, Paris, France; ^2^INSERM, UMRS1138, Team 17, From physiopathology of ocular diseases to clinical development, Sorbonne Paris Citeí University, Centre de Recherche des Cordeliers, Paris, France; ^3^Borelli Centre, UMR 9010 CNRS—SSA—ENS Paris Saclay—Paris Cité University, Paris, France

**Keywords:** gene therapy, children, voretigene neparvovec, pediatric ophthalmology, genetic ocular diseases, aniridia, optogenetic therapy, ataluren

## Abstract

Genetic pediatric eye disease frequently leads to severe vision impairment or blindness. Voretigene neparvovec is the first approved gene therapy for an inherited retinal dystrophy (IRD). Voretigene neparvovec has been shown to be well tolerated and safe, with encouraging results in terms of efficacy, mainly when administered early in childhood. While we assisted at the first gene therapy available in clinical practice for an IRD, some questions remain unanswered, especially when gene therapy is delivered in young children. We review here the most recent reports and promising ongoing studies concerning various approaches on gene therapy in pediatric ophthalmology.

## 1 Introduction

Genetic pediatric eye disease frequently leads to severe vision impairment and/or blindness, with long-lasting individual and societal impacts. Gene therapy involves the transfer of genetic material to remove, replace, repair, or introduce a gene, or to overexpress a protein, which has a therapeutic impact. Even though for most anterior segment diseases gene therapy still relates to the preclinical stage (1, 2), for inherited retinal disorders translation has been reached. Over the last 10 years, gene therapy for biallelic *RPE65*-mediated inherited retinal dystrophy has been the subject of many clinical trials, leading to the first United State Food and Drug Administration (US FDA)-approved ocular gene therapy for the treatment of an inherited retinal disorder. Voretigene neparvovec (VN, Luxturna^®^), administered by subretinal injection after a 25-gauge vitrectomy, uses a non-replicating adeno-associated virus (AAV) as a vector to transfer a functional copy of the *RPE65* gene into the retinal pigment epithelium cells. VN has been shown to be well tolerated and safe in humans (3–7), with encouraging results in terms of efficacy, mainly when administered early in childhood (8–12).

## 2 Voretigene neparvovec for pediatric patients with biallelic *RPE65*-mediated inherited retinal dystrophy

A Phase III study reported in 2017 led to US FDA and European Medicines Agency (EMA) approval of VN (9). The study included 20 treated patients and nine control patients; 40% of patients were aged 3 to 10 years. The primary endpoint was based on the results of the multi-luminance mobility testing (MLMT) score change from baseline to year 1. MLMT had been designed by Spark Therapeutics to measure changes in functional vision, as assessed by the ability of a subject to navigate a mobility course accurately in different levels of environmental illumination. A significant change in MLMT score (1.8 *vs*. 0.2 light levels, p = 0.0013) was reported when comparing 20 treated patients with nine control patients. In addition, 65% of treated patients (13/20), but no control patients, passed the MLMT at the lowest level of luminance (1 lux, equivalent to a summer night with a full moon). The mean improvement in BCVA was not significant between groups (+8.1 letters in treated subjects *vs*. +1.6 letters in control participants). However, a significant improvement was reported in the treated group compared with the control group for full-field light sensitivity threshold (FST), Goldmann visual field (VF, III4e), and macular sensitivity threshold (Humphrey). Reported adverse effects included elevated intraocular pressure (20%), cataracts (15%), retinal tears (10%), eye inflammation (10%), macular holes (5%), maculopathy (5%), and retinal hemorrhages (5%). More recently, it has been reported that improvements in ambulatory navigation, light sensitivity, and visual field lasted for at least 3–4 years (10).

Weleber et al. have suggested that the greatest improvements in visual acuity ranged from 6 to 14 letters in pediatric patients (aged 6–11 years) at 2 years (11). More recently, Sengillo et al. reported significant vision change that reached statistical significance only in the pediatric population, with a mean follow-up of 10 months (13). Testa et al. have reported six consecutive pediatric patients treated bilaterally with VN. Significant improvement of BCVA and outer nuclear layer thickness of the internal ETDRS‐ring on SD-OCT at day 30/45 and day 180 were shown, suggesting that improvement of visual acuity could be related to partial recovery of retinal morphology in the perifoveal ring (14). At 6 months a mean change of −0.2 logMAR (SD ± 0.07 logMAR) was observed. In particular, six eyes (50%) showed an improvement of one ETDRS line and the remaining six eyes (50%) improved by two ETDRS lines. Interestingly, intra‐operative foveal detachment was not associated with a higher function gain in terms of BCVA. Deng et al. reported a follow-up of 27 eyes of 14 pediatric patients (age range 4–17 years) with a mean visual acuity improvement of +7.5 to +12.5 ETDRS letters (15). Additionally, significant improvements in FST and Goldmann visual fields were reported for all patients. Elevation in intraocular pressure (59%), persistent intraocular inflammation (15%), and vitreous opacities (26%) were the most important side effects. Gerhardt et al. reported in four young children (aged 3–6 years, eight eyes) a marked increase in vision-guided behavior and in visual acuity, with a mean change of >0.30 logMAR units 6 months after gene therapy (16). Two eyes showed partial electrophysiological recovery of an ERG that was undetectable before treatment. Intra‐operative foveal detachment was reached in all eyes. Rhegmatogenous retinal detachment was reported in two eyes and circumscribed atrophy of the photoreceptor layer at the injection site was reported in one eye.

Development of subretinal deposits has been observed at 1 week post-operatively in three pediatric patients (ages: 22 months, 2 years, and 5 years) (17). All three patients experienced improved visual function and subretinal deposits improved or resolved over the follow-up period.

A previously undescribed perifoveal chorioretinal atrophy was observed in 18 eyes of 10 patients (mean age 11.6 years, range 5–20 years) who underwent post-market VN treatment by subretinal injection (18). The perifoveal chorioretinal atrophy was first noticeable at an average of 4.7 months (SD 4.3 months) following surgery, and progressively enlarged in all cases up to the last follow-up examination (mean follow-up at 11.3 months). Despite this atrophy, improvements in visual acuity, FST, and visual field were evidenced in the majority of patients. It is important to highlight that atrophy was observed within and outside the area of the subretinal bleb in 10 (55.5%) eyes, exclusively within the area of the bleb in seven (38.9%) eyes, and exclusively outside the bleb in one (5.5%) eye. Further studies are necessary to determine whether ocular conditions, surgical delivery, and/or vector-related parameters predispose patients to this complication, and to determine the way it evolves in the long term.

## 3 Other gene therapies for pediatric patients with retinal inherited dystrophies

Various promising approaches have been investigated for retinal inherited dystrophies, including antisense oligonucleotides (AON), CRISPR-based genome editing techniques, and mutation-independent strategies.

### 3.1 Leber congenital amaurosis

An intravitreal injection of AON, which induces persistent suppression of pathological RNA transcripts by exon skipping, has shown improvement in visual acuity at 3 months without serious adverse effects in *CEP290*-Leber congenital amaurosis (19, 20). A Phase I/II clinical trial has recently shown a manageable safety profile and improvements in visual acuity and retinal sensitivity, supporting the continuation of AON (QR-110, sepofarsen) development (21). A Phase II/III double-masked, randomized, controlled, multiple-dose study evaluating the efficacy, safety, tolerability, and systemic exposure of intravitreal injections of AON (QR-110) in *CEP290*-Leber congenital amaurosis has failed to meet the primary endpoint (ClinicalTrials.gov identifier: NCT03913143). However, *post hoc* analyses reveal multiple pointers of a beneficial effect when comparing sepofarsen with a sham group if the contralateral eyes in each group are adjusted for (22). Additional analyses are ongoing (22).

An innovative CRISPR-based genome editing technique is an exciting approach currently being evaluated for *CEP290*-Leber congenital amaurosis (23). The ongoing Phase I/II clinical trial evaluates the safety, tolerability, and efficacy of single escalating doses of EDIT-101, a novel gene editing product designed to eliminate the mutation on the *CEP290* gene, administered *via* a subretinal injection (ClinicalTrials.gov identifier: NCT03872479).

A Phase I/II clinical trial evaluating the safety and tolerability of ascending doses of SAR439483 administered as subretinal injections in patients with Leber congenital amaurosis caused by autosomal recessive guanylate cyclase 2D (GUCY2D) mutations (GUCY2D-LCA) is ongoing (ClinicalTrials.gov identifier: NCT03920007).

### 3.2 X-linked retinitis pigmentosa

A single, subretinal injection of cotoretigene toliparvovec (BIIB112/AAV8-RPGR) gene therapy has shown an early and sustained improvement in retinal sensitivity and low-luminance visual acuity in some participants through 12 months, supporting consideration of additional clinical trials for gene therapy in X-linked retinitis pigmentosa (RP) (24, 25).

Three Phase III clinical trials including children are ongoing to determine the efficacy of a subretinal administration of AAV5-RPGR (ClinicalTrials.gov identifier: NCT04671433), a subretinal administration of rAAV2tYF-GRK1-hRPGRco (ClinicalTrials.gov identifier: NCT04850118), and an intravitreal injection of 4D-125 in patients with X-linked RP.

### 3.3 Achromatopsia

An open-label, non-randomized controlled trial has shown that gene therapy vector AAV8.CNGA3 administered by a subretinal injection in adult patients with achromatopsia had no substantial safety problems and had visual acuity and contrast sensitivity gains (26), paving the way for an achromatopsia gene therapy at a younger age. This is particularly important because the lack of cone photoreceptor input at a young age can have long-term effects on the development of the visual cortex, limiting the benefits of such gene therapy in adult patients (27, 28). Several Phase I/II clinical trials to examine AAV therapies in both children and adult patients with CNGB3 and CNGA3 achromatopsia are ongoing (ClinicalTrials.gov identifiers: NCT03001310, NCT03758404, NCT02935517, NCT02599922).

### 3.4 X-linked retinoschisis

An open-label, Phase I/II dose-escalation clinical trial evaluated the safety and efficacy of rAAV2tYF-CB-hRS1, a recombinant adeno-associated virus vector expressing retinoschisin (RS1), in 22 adults and five children with retinal disease caused by mutations in the *RS1* gene. The therapy was generally safe and well tolerated but failed to demonstrate a measurable treatment effect. The clinical trial is ongoing through 5 years of follow-up in order to assess its long-term safety (29).

### 3.5 Mutation-independent strategies

Various congenital disorders already exhibit severe developmental defects or cell loss at birth, limiting the potential for viral gene therapy. Thus, mutation-independent strategies seem promising to maintain cell survival or restore visual function. Optogenetic therapies deliver light-activated ion channels to surviving retinal cell types (for instance, bipolar cells and retinal ganglion cells), restoring photosensitivity. Partial functional recovery has recently been reported in an adult patient with advanced non-syndromic rod-cone retinal dystrophy after optogenetic therapy (30). The treatment combined the injection of an optogenetic vector and the use of light-stimulating goggles. The patient was able to perceive, locate, count, and touch various objects using the vector-treated eye alone while wearing the goggles. A Phase I/II clinical trial for adults with non-syndromic retinal dystrophy is ongoing (ClinicalTrials.gov identifier: NCT03326336).

Multi‐characteristic opsin (MCO) has been shown to effectively re‐photosensitize photoreceptor‐degenerated retina in mice, leading to vision restoration in an ambient light environment (31). A Phase IIB randomized, double-masked, sham-controlled study to evaluate the efficacy and safety of intravitreal injections of vMCO-010 optogenetic therapy in adults with advanced RP is ongoing (ClinicalTrials.gov identifier: NCT04945772).

Other mutation-independent strategies tested in mouse models of retinal degeneration aimed to promote photoreceptor cell survival, as a CRISPR-mediated knockdown of the key transcription factor Neural Retina Leucine zipper (Nrl), or a viral-mediated expression of the rod-derived cone viability factor (RdCVF) (32, 33). Whether these strategies will translate into long-lasting restoration of retinal function in humans remains to be determined.

## 4 Congenital aniridia

Congenital aniridia (OMIM# 106210) is a rare panocular malformation caused by loss of function variants in the Paired box 6tbox6 gene (*PAX6*) or 11p13 chromosome rearrangements, and more than 700 pathogenic variants have been reported to date (34). Ataluren, a nonsense mutation suppression therapy, enables a ribosomal read-through of mRNA-containing premature termination codons, resulting in the production of a full-length protein. Postnatal administration of ataluren eye drops reverses congenital tissue malformation defects in Pax6^Sey+/−^ mice (35). A Phase II clinical trial evaluating oral ataluren, which has already been approved for the treatment of Duchenne muscular dystrophy, failed to meet its primary endpoint (ClinicalTrials.gov identifier: NCT02647359). An ophthalmic formulation of ataluren has been recently assessed (36), opening new therapeutics perspectives in congenital aniridia.

## 5 Discussion

Restoring vision in children remains a challenge in ophthalmology. Gene therapy opens a promising field and is in constant development. The number of clinical trials for retinal gene therapies has increased dramatically over the last decade. While we assisted with the first gene therapy available in clinical practice for an IRD, some questions remain unanswered, especially when gene therapy is delivered in young children.

It has been suggested that better outcomes are observed when Luxturna^®^ is administered earlier in childhood. However, assessment of efficacy in very young children is not always feasible and remains to be determined. Early treatment indications should then balance phenotype and disease progression, patient cooperation to assess functional quantifiable measures (visual acuity, visual field, full-field ERG, FST), and potential treatment-related complications. Subretinal injections in pediatric patients’ eyes could result in several complications that could interfere with visual outcome and should therefore be performed by experienced pediatric vitreoretinal surgeons. Whether foveal detachment should be induced remains a key unsolved aspect. Finally, little is known about long-term durability (beyond 4 years), which is essential in the pediatric population.

The administration route plays an important role in the success of gene therapy. In congenital aniridia, oral ataluren failed to meet its primary endpoint, apparently due to patient age and phenotypical discrepancies. However, results could possibly be different if a topical route of administration were used, improving availability of the drug for the eye and cornea (34). The recent development of a drop formulation of ataluren (36) should encourage clinical trials in congenital aniridia.

A growing number of innovative therapies are the objects of ongoing clinical trials and these have the potential to become a standard of care for IRD within the next few years. To take full advantage of advances in gene therapy, it is critical to improve early diagnosis of IRD and facilitate genetic identification from a young age. Rare disease centers of excellence with the infrastructure and expertise for precise phenotyping and clinical networks represent the basis of an organizational structure that ensures access to care for patients and phenotype characterization of rare diseases. A multidisciplinary collaboration at national and international levels among clinicians, molecular biologists, clinical geneticists, and clinical and scientific researchers should be promising in terms of improving the diagnosis, treatment, and follow-up of this rare and complex disease, in addition to including patients in national registries in order to contact them when mutation-specific therapies are becoming available.

## Author contributions

AD and DB-G contributed to the conception of the study and literature review. AD wrote the first draft of the manuscript. MR and DB-G wrote sections of the manuscript. All authors contributed to the article and approved the submitted version.
